# Polycaprolactone/Doped Bioactive Glass Composite Scaffolds for Bone Regeneration

**DOI:** 10.3390/jfb16060200

**Published:** 2025-06-01

**Authors:** Ana Sofia Pádua, Manuel Pedro Fernandes Graça, Jorge Carvalho Silva

**Affiliations:** 1 CENIMAT|i3N, Department of Materials Science, School of Science and Technology, Nova University Lisbon, 2829-516 Caparica, Portugal; as.padua@campus.fct.unl.pt; 2 i3N and Physics Department, Aveiro University, 3810-193 Aveiro, Portugal; mpfg@ua.pt; 3 CENIMAT|i3N, Department of Physics, School of Science and Technology, Nova University Lisbon, 2829-516 Caparica, Portugal

**Keywords:** bioactive glass, tantalum, copper, zinc, magnesium, niobium, polycaprolactone, composites, bone tissue engineering

## Abstract

Critical-size bone defects do not heal spontaneously and require external support, making bone regeneration a central challenge in tissue engineering. Polymeric/ceramic composite scaffolds offer a promising approach to mimic the structural and biological properties of bone. In this study, we aimed to evaluate the effect of different doping oxides in bioactive glass (BG) on the performance of polycaprolactone (PCL)-based composite scaffolds for bone tissue engineering applications. Composite scaffolds were fabricated using solvent casting, hot pressing, and salt-leaching techniques, combining PCL with 25 wt% of BG or doped BG containing 4 mol% of tantalum, zinc, magnesium, or niobium oxides, and 1 mol% of copper oxide. The scaffolds were characterized in terms of morphology, mechanical properties, and in vitro biological performance. All scaffolds exhibited a highly porous, interconnected structure. Mechanical compression tests indicated that elastic modulus increased with ceramic content, while doping had no measurable effect. Cytotoxicity assays confirmed biocompatibility across all scaffolds. Among the tested materials, the Zn-doped BG/PCL scaffold uniquely supported cell adhesion and proliferation and significantly enhanced alkaline phosphatase (ALP) activity—an early marker of osteogenic differentiation—alongside the Nb-doped scaffold. These results highlight the Zn-doped BG/PCL composite as a promising candidate for bone regeneration applications.

## 1. Introduction

Bone is known for its remarkable self-healing capacity, enabling the complete reconstruction of tissue while restoring its original properties and morphology [1,2]. However, when critical-size defects (equal to or larger than 2.5 cm) occur due to trauma, tumor removal, infection, genetic abnormalities, or metabolic disorders, the endogenous regenerative capacity of bone tissue is impaired, requiring additional support [3,4]. Consequently, the gold-standard treatment for large bone defects is autologous bone grafting, in which the patient’s own tissue is harvested from a healthy site [5,6]. However, the limited availability of donor tissue and the harvesting procedure itself can lead to various complications, including infection, chronic pain, blood loss, nerve injury, trauma, and, in extreme cases, morbidity [7,8]. These challenges have driven increased research into the development of biomaterials for bone regeneration, from platelet-rich fibrin (PRF) [9] to composites that mimic bone morphology [10].

Bioactive glass (BG) is one of the most widely studied biomaterials due to its outstanding features, including bioactivity [11], biodegradability [11,12], the ability to promote osteogenesis [12,13] and angiogenesis [14], as well as anti-inflammatory [15,16] and immunomodulatory properties [16,17]. These features make BG highly suited for bone regeneration. However, traditional BGs have unsatisfactory handling properties, low plasticity, poor rapid-setting ability, and low space-making ability, which limits their applications [18,19,20,21,22]. To overcome these limitations, incorporating BGs into composite scaffolds with a macroporous structure that facilitates implantation while preserving their therapeutic properties is a promising strategy [19,20]. For this purpose, various scaffolds containing 45S5 BG and resorbable synthetic polymers, such as polycaprolactone (PCL), have been developed using different fabrication techniques, including 3D printing, electrospinning, lyophilization, and salt leaching [23,24,25,26]. A balance between mechanical stability, bioactivity, and biological response is important in scaffolds intended for load-bearing bone regeneration. The scaffold must not only provide sufficient mechanical support to withstand physiological loads and prevent structural failure before new bone is formed, but also be bioactive to promote cell adhesion, proliferation, and osteogenic differentiation, typically through ionic release or surface interactions. These mechanical and biochemical cues work together to direct a favorable biological response, proper integration with host tissue, and successful bone regeneration.

Some techniques can control the final geometry of the scaffold, such as 3D printing [27,28,29]. One composite fabricated using this technique is a mesoporous bioactive glass (MBG) composed of SiO_2_-CaO-P_2_O_5_, combined with a polycaprolactone (PCL) scaffold (MBG-PCL). This scaffold demonstrated good biocompatibility in vitro when in contact with osteoblasts and osteoclasts. In an in vivo osteoporotic sheep model, the scaffold exhibited excellent bone regeneration, with high vascularization, promotion of new bone formation throughout the scaffolds, thick trabeculae, and a high presence of osteoblasts and osteoclasts [30]. Furthermore, this scaffold enhanced colonization throughout the structure by dissolving chemical signals and forming hydroxyapatite (HAp), which increased pre-osteoblast proliferation and differentiation, as indicated by the upregulation of alkaline phosphatase (ALP) expression. The architectural features of the scaffolds also influenced cell migration, as migration routes within the scaffolds depend on the interconnections between layers [31].

Furthermore, variations in BG content can modulate different responses. The 3D printed 58S BG/PCL scaffold exhibited increased cell adhesion and proliferation with higher BG content. Additionally, a higher BG content upregulated the expression of runt-related transcription factor 2 (RUNX2) and collagen type I (Col I). In an in vivo radial defect repair model in rats, bone repair was also enhanced with increasing bioactive glass content in the scaffold. The composite scaffold with the highest BG content (20 wt%) showed the greatest improvement in bone repair [32].

However, the incorporation of BGs alone is not sufficient to address recent challenges in biomaterials science and tissue engineering, such as cancer treatments and antibiotic-resistant microorganisms. To tackle these issues, research has increasingly focused on incorporating various therapeutic inorganic ions (TIIs) into BG systems to impart specific biological functionalities and enhance their therapeutic performance [14], including antibacterial [33,34,35], haemostatic [36,37], and immunomodulatory properties [38,39].

Copper is a TII known for its angiogenic, vasculogenic, and antibacterial properties. Due to these properties, Cu doping has already been used in several composites, including nanofibrillated cellulose (NFC) composite membranes and aerogels [40], Cu-containing BG/eggshell membrane composites [41], and collagen-Cu-doped BG scaffolds (CuBG-CS) [42]. However, this doping agent exhibits a dose-dependent response, making it safe only at low concentrations [43,44].

In a study on doped BG, Sergi et al. presented a comparative analysis of PCL/BG composite nanofiber mats containing 45S5, Sr- and Mg-substituted BG, or Zn-, Sr-, and Mg-substituted BG. All doped composite electrospun mats demonstrated increased mechanical strength. However, bioactivity and biological response analyses showed that Sr/Mg-substituted BG had higher HAp precipitation as well as increased cell proliferation and migration rates, indicating that this scaffold has a higher wound-healing potential [45].

Using 3D printing technology, Fathi et al. produced Sr- and Co-doped BGs combined with PCL. This multi-component scaffold demonstrated potential as a bone replacement due to its compressive strength, hydrophilicity, bioactivity, and good cytocompatibility. Moreover, the scaffolds served as suitable substrates for the adhesion and proliferation of osteosarcoma cells (MG-63). Overall, the best compromise between mechanical stability and biological properties was achieved with a 40 wt% BG content [46]. Additionally, the incorporation of SiO_2_:SrO:P_2_O_5_ into PCL/MBG composite thin membranes enhanced bioactivity, as well as mechanical properties such as elastic modulus and hardness, and improved osteogenic potential. However, this study did not allow for the identification of a composite with significantly superior characteristics compared to the others [47].

The incorporation of different Co- and Mg-co-doped 45S5 BG into PCL/BG electrospun composite membranes showed no cytotoxic response in fibroblasts. Among the tested doping agents, an increase in Co content enhanced VEGF expression, while an increase in Mg content slightly upregulated HIF-1α expression. These results confirm the angiogenic properties of Co [48].

In previous studies, 2 mol% Zn-doped BG and 45S5 BG were incorporated into porous PCL membranes. The results showed that zinc addition promoted cell viability and increased ALP production [49]. However, other studies suggest that a higher ZnO concentration could further enhance BG cell responses [50,51].

No studies have yet investigated the effect of doping TII elements, such as tantalum or magnesium, in a composite material with PCL that mimics spongy bone for regeneration applications. Therefore, the main objective of this work was to fabricate and characterize composite scaffolds with PCL and BG doped with various ions, including tantalum, copper, zinc, magnesium, and niobium. Scaffolds were produced using solvent casting, hot pressing, and salt-leaching techniques. These composites were structurally and biologically characterized and compared to PCL scaffolds and undoped 45S5 BG/PCL scaffolds to identify the most promising BG doping agent for bone regeneration applications.

## 2. Materials and Methods

### 2.1. Preparation of PCL and BG/PCL Scaffolds

The composite scaffolds were fabricated using solvent casting, hot pressing, and salt-leaching techniques. The doping level of BG and the BG concentrations used in the scaffolds were chosen based on the results of previous studies reported by Gavinho et al. [49,52] and Hammami et al. [43,53] Because both Cu [43] and Mg [52] pose a risk of cytotoxicity at high concentrations, the ceramic content in the scaffolds was reduced to 10% for Cu and 20% for Mg.

The BG powders were synthesized using the melt-quenching method, following the 45S5 Bioglass^®^ formulation (46.1SiO_2_-2.6P_2_O_5_-24.35Na_2_O-26.91CaO, mol%) proposed by L. Hench. For the doped BG variants, five compositions were developed by incorporating different ions, including tantalum, copper, zinc, magnesium, and niobium oxides, into the 45S5 BG formulation. The BG powders were passivated by submerging the powders in water for 24 h, then removing the water and drying the powders before scaffold production.

PCL (Sigma-Aldrich, Algés, Portugal) was dissolved in chloroform at a constant concentration of 10 wt%, with NaCl particles (selected by sieving with 100 µm and 200 µm meshes) added at 5 wt%. The composite mixtures contained varying concentrations of 45S5 BG, with and without doping ions, depending on the specific BG used. Table 1 summarizes all the solutions prepared for scaffold synthesis.

The mixtures were placed on a magnetic stirrer and stirred vigorously throughout the night to achieve a uniform slurry. These mixtures were then poured into films, from which disks with a 20 mm diameter were cut. The polymeric and composite scaffolds were created using a hot platen press at 60 °C with a pressure of 3 tons for 5 min. The salt was then removed by soaking the samples in distilled water with continuous stirring for 24 h, after which they were dried.

### 2.2. Morphological Characterization

The surface morphology of the sample was evaluated using a Regulus 8220 field emission scanning electron microscope (FE-SEM) from Hitachi (Tokyo, Japan). The scaffolds were mounted on aluminum platforms for horizontal viewing and sputter-coated with a gold–palladium conductive layer using a Q300T D sputter coater (Quorum Technologies, Laughton, UK). A semi-quantitative analysis of the samples’ chemical composition was performed using a INCA Energy 350 Energy Dispersive Spectroscopy (EDS) system from Oxford Instruments (High Wycombe, UK) coupled to the microscope.

### 2.3. Chemical Characterization

For chemical characterization, attenuated total reflectance Fourier-transform infrared (ATR-FTIR) spectroscopy was performed using a PerkinElmer spectrometer (Waltham, MA, USA). This technique analyzes the infrared absorption of chemical bonds within the molecules of the sample constituents. Spectra were recorded over a frequency range of 4000 to 500 cm⁻^1^, with a resolution step size of 1 cm^−1^.

### 2.4. Mechanical Characterization

The mechanical properties of the scaffolds were determined using a Texture Lab testing machine (Food Technology Corporation, Braintree, UK), which was fitted with a 250 N load cell. The tests were conducted at a crosshead speed of 1 mm/min in compression mode and at room temperature. The compression modulus was determined from the slope of the stress–strain curve within the linear region of the elastic regime using OriginPro v2018 software (OriginLab, Northampton, MA, USA). This assay included at least five replicates for each sample.

### 2.5. Cell Culture

#### 2.5.1. Cytotoxicity Assay

Cytotoxicity assays were performed according to the ISO 10993-5 standard [54] using the extract method. Samples were sterilized with 70% ethanol for 20 min, dried for 48 h, and then placed in contact with the cell culture medium (McCoy’s 5A) for 48 h at an initial concentration of 100 mg/mL. Saos-2 cells (ATCC^®^ HTB-85™) were seeded at a concentration of 30,000 cells/cm^2^ in a 96-well plate and incubated for 24 h. Afterwards, the medium was replaced with the filtered extracts and their respective dilutions, using four replicates for each condition. The extracts were incubated for 48 h, after which cell viability was measured using a resazurin absorbance test. For this test, the extracts were replaced with a resazurin solution at 0.02 mg/mL, and after 3 h of incubation, absorbance was read using an ELx 800UV microplate reader from Biotek (Winooski, VT, USA). This test was standardized using a negative control (cells cultured in a standard, non-cytotoxic environment) and a positive control (cells exposed to a cytotoxic environment created by medium containing 10% DMSO, a cytotoxic agent).

#### 2.5.2. Adhesion and Proliferation

The ability of the scaffolds to support cell metabolism was evaluated through cell adhesion and proliferation studies.

The scaffolds were sterilized as previously described in the cytotoxicity assays. Afterwards, the samples were placed in 24-well plates, fixed with silicone O-rings, and covered with McCoy’s medium. The cell control consisted of a glass cover slip, also fixed with an O-ring. Prior to cell culture, the medium was aspirated, and osteoblast-like cells (Saos-2) were plated at a density of 30,000 cells/cm^2^ directly onto the sample surface. Control cells were seeded onto glass coverslips. Cells were kept in McCoy’s medium and incubated for 24 h at 37 °C in an environment with 5% CO_2_.

The cell adhesion rate was determined by the reduction of resazurin, as in the cytotoxicity tests. For this process, the medium was replaced with a 1:1 solution of resazurin (diluted to a concentration of 0.04 mg/mL in PBS) and McCoy’s medium, followed by incubation for 3 h. The incubated media were then transferred to a 96-well plate for absorbance measurement at 570 nm and 600 nm using a microplate reader (Biotek ELx 800UV). The resazurin assay was repeated on days 3, 7, 10, and 14 to assess the cell population on each material. Cell proliferation was then calculated as the ratio between day 1 and day 14 measurements.

This assay included three technical replicates for each of the three independent biological replicates.

#### 2.5.3. Alkaline Phosphatase (ALP) Activity

ALP is an enzyme produced by cells during osteogenesis and is widely recognized as a differentiation marker [55]. To measure ALP expression, a colorimetric assay was employed, which involved analyzing the increase in absorbance at 405 nm. The reaction utilized 1 mg/mL of 4-nitrophenyl phosphate disodium salt (Sigma-Aldrich), which was dissolved in a Tris-hydrochloric acid solution.

The assay involved filtering the media that had been in contact with the samples to remove dead cells and cell debris, as well as with the cell and blank controls, which included both the material and the medium. The baseline was obtained by measuring the initial absorbance of the filtered solutions. Then, the pNPP solution was added to the media at a 1:1 ratio and incubated for 20 min. Absorbance was then measured and normalized to the previous day cell population.

#### 2.5.4. Immunofluorescence Staining

For immunofluorescence staining, the samples were washed three times with PBS and fixed with 4% PFA for 20 min at the end of the 15-day culture period. Afterwards, they were washed with PBS, permeabilized with 0.2% Triton X-100 in PBS for 15 min, and washed again with PBS. Actin staining was performed by incubating the cells with a phalloidin conjugate (CoraLite^®^594-Phalloidin, Proteintech, Rosemont, IL, USA) diluted in PBS at a 1:400 ratio for 40 min in the dark. DNA was then counterstained with 10 µg/mL DAPI in PBS for 5 min in the dark at room temperature. To clean the cells, the coverslips were washed three times with PBS for 5 min each (500 µL/well), rinsed with Milli-Q water, and mounted on slides using Mowiol (Sigma-Aldrich) as the mounting medium. Cell nuclei and F-actin were visualized using an Eclipse Ti-S fluorescence microscope (Nikon, Tokyo, Japan), which was paired with a Nikon D610 digital camera. The images were captured with a 40× objective lens.

### 2.6. Statistical Analysis

The in vitro results were statistically analyzed using OriginPro 2018 software and are expressed as mean ± standard deviation. Furthermore, statistical analysis was conducted through one-way analysis of variance (One-way ANOVA) with Tukey’s multiple comparison test at *p* values of 0.05, 0.01, and 0.005. Results with *p* < 0.05 were deemed statistically significant.

## 3. Results and Discussion

### 3.1. Chemical Characterization

**Figure 1:**
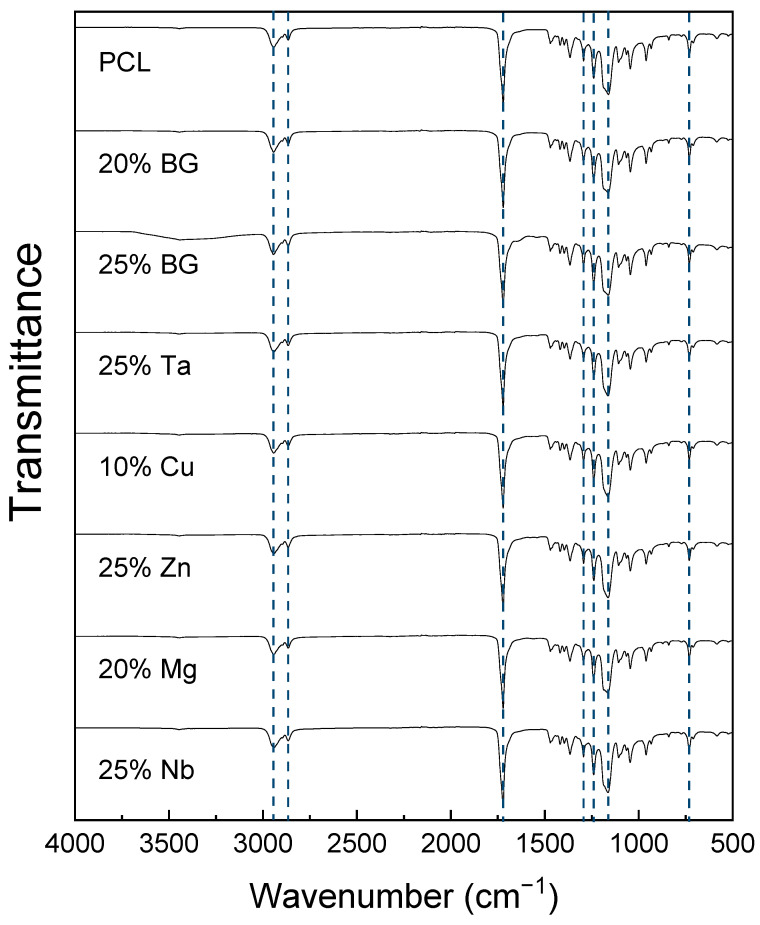
FTIR analysis of the PCL and composite scaffolds, with both undoped and doped BG.

The FTIR spectrum of the PCL scaffold, presented in Figure 1, exhibited characteristic absorption bands of PCL [32,45]. The peaks at 2944 cm^−1^ and 2866 cm^−1^ correspond to the asymmetric and symmetric stretching vibrations of CH_2_ groups, respectively [56]. The peak at 1721 cm^−1^ was attributed to the C=O stretching vibration of the carbonyl group [25]. The band at 1294 cm^−1^ was associated with C-O and C-C stretching vibrations, while the peaks at 1240 cm^−1^ and 1162 cm^−1^ correspond to the asymmetric and symmetric stretching vibrations of the C-O-C groups, respectively [57]. Additionally, the peak at 732 cm^−1^ was attributed to the CH_2_ long chain rocking motion vibrations [58].

The studied composites all present the PCL peaks and bands. However, none of the fingerprint bands of BG, such as Si-O-Si bands (symmetric stretching at 850 and asymmetric stretching mode at 1010 cm^−1^) and Si-O bands (910 cm^−1^ and 730 cm^−1^) were detected [59,60,61]. The signal may have been suppressed due to the surface sensitivity of the ATR-FTIR technique and the BG particles being encapsulated in the PCL matrix.

### 3.2. Morphological Characterization

**Figure 2:**
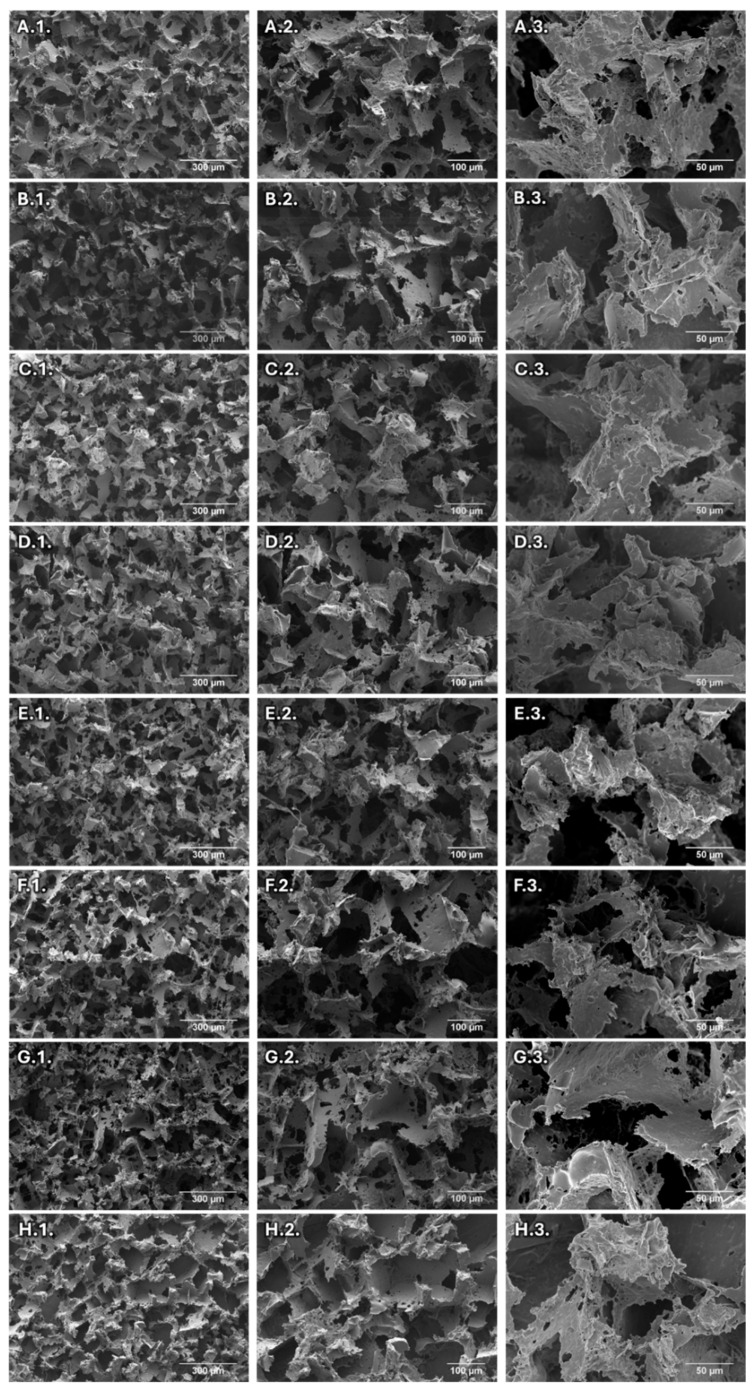
SEM images, with several magnifications (100×, 200× and 500×, identified with the numbers 1, 2 and 3 in the images), of the PCL scaffolds (**A**), PCL:20BG (**B**), PCL:25BG (**C**), PCL:25TaBG (**D**), PCL:10CuBG (**E**), PCL:25ZnBG (**F**), PCL:20MgBG (**G**) and PCL:25NbBG (**H**).

The scaffold morphology can be analyzed in the SEM images presented in Figure 2. All manufactured scaffolds present an interconnected porous structure. The scaffolds fabricated in this work using chloroform have a similar morphology to the samples prepared with acetone, as previously reported by Gavinho et al. [49]. Furthermore, the addition of BG and doped BG to the composite did not affect the microstructure or porosity of the scaffolds, as all scaffolds feature both macro and micro porosity. The macro porosity predominantly consists of pores with diameters between 100 and 200 μm, which is attributable to the size of the NaCl particles used. Additionally, the hot pressing process, where both the NaCl particles and melted polymer are compacted, promotes contact between the salt grains, creating interconnectivity between the pores,. This interconnectivity and porous morphology are ideal for cell adhesion, proliferation, and angiogenesis as well as the integration of the scaffolds with surrounding tissue, thereby enhancing the mechanical stability of the implant [62,63,64].

**Figure 3:**
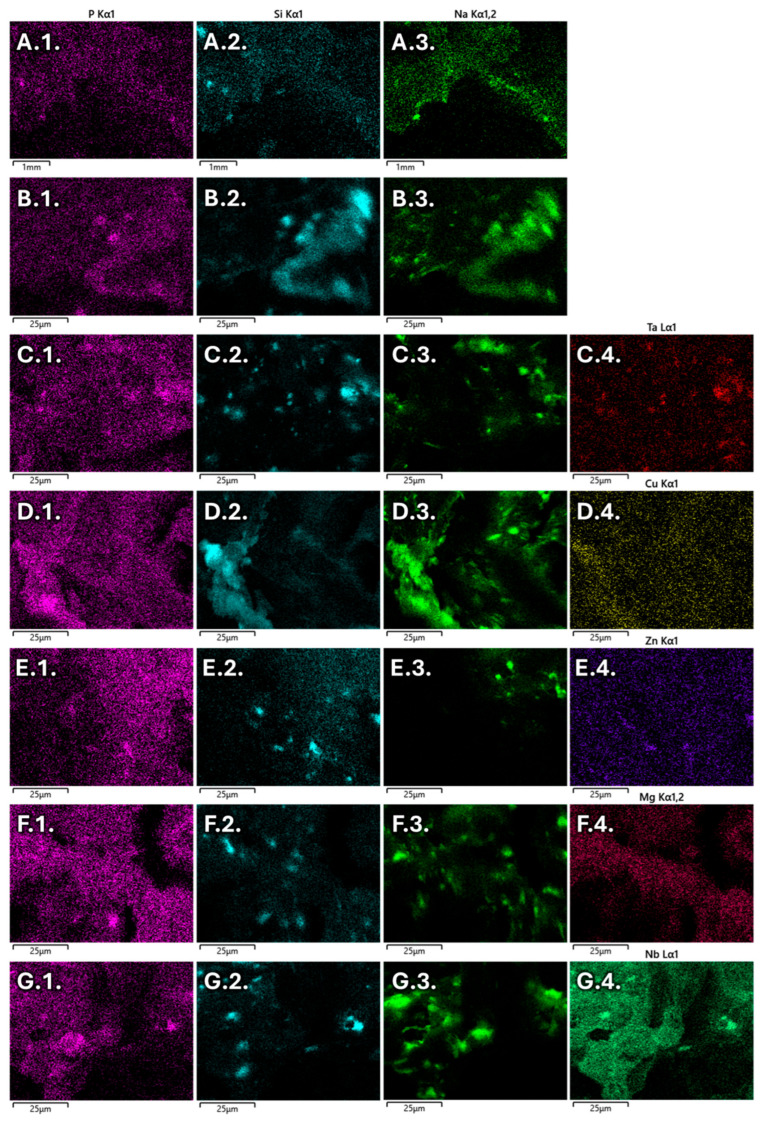
EDS mapping of 20% BG/PCL sample (**A**), 25% BG/PCL sample (**B**), 25% Ta/PCL sample (**C**), 10% Cu/PCL sample (**D**), 25% Zn/PCL sample (**E**), 20% Mg/PCL sample (**F**) and 25% Nb/PCL sample (**G**).

The EDS analysis presented in Figure 3 confirms the presence of BG in all samples due to the detection of BGs components, such as phosphorus (column 1), silicon (column 2), and sodium (column 3). Furthermore, the doping agent is confirmed in column 4, where their distribution is presented.

The BG distribution is generally homogeneous within the scaffold; however, some particle aggregation occurred due to the passivation process and subsequent drying of the BG powders. The increase in BG concentration in the scaffolds also contributed to greater BG powder aggregation.

### 3.3. Compression Modulus

A key objective in bone tissue engineering is the development of scaffolds that combine high porosity with sufficient mechanical strength to support load-bearing applications. However, increased porosity often compromises mechanical integrity [65]. Therefore, there is a critical need for biomaterials that maintain adequate strength while supporting the highly porous structures favored in tissue engineering.

**Figure 4:**
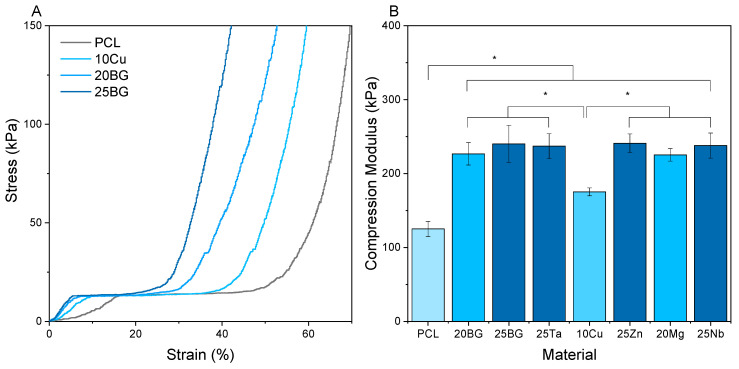
Compression tests of PCL and composite scaffold samples. (**A**) Stress–strain curves and (**B**) elastic compression modulus. Significant differences were noted between the samples (* *p* < 0.05).

To evaluate the compression modulus of the porous composite scaffolds, each sample was cut into a cylinder with a diameter of 7 mm and tested under compressive loading. The resulting load–displacement data, presented in Figure 4A), revealed consistent stress–strain patterns across all scaffolds. The patterns displayed three distinct phases: an initial linear elastic region, a yield point leading to a collapse plateau, and finally, densification regimes [66,67]. The inclusion of BGs in the scaffolds resulted in an increase in the elastic slope during the initial 15% of the stress–strain curve, due to the ceramic filler’s reinforcement effect.

The compression modulus results presented in Figure 4B) indicate that, for a given BG content, all samples exhibit a similar compression modulus regardless of the doping agent [68,69]. The compression modulus increases with the concentration of BGs incorporated in the composites. This increase follows a linear pattern from the PCL scaffold to the 20% BG content in the scaffolds. However, the mechanical enhancement with higher ceramic content diminishes when 25% BG is added, as there is no significant difference between the 20% BG and 25% BG scaffolds. This phenomenon can be attributed to the aggregation of BG particles at 25%.

### 3.4. Cell Culture

Cell responses to composite scaffolds containing doped and undoped bioactive glasses were evaluated using cytotoxicity, adhesion, and proliferation assays.

#### 3.4.1. Cytotoxicity Assay

**Figure 5:**
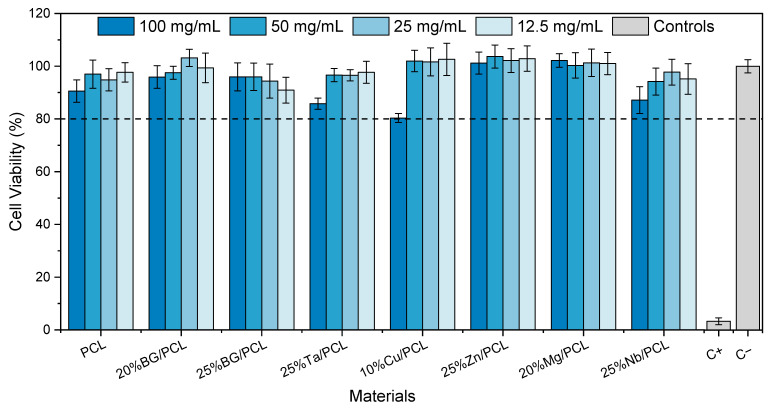
Relative osteoblast-like cells viability after 48 h incubation with PCL scaffolds and composite scaffolds with undoped 45S5 BG doped BGs extracts. C+ is the positive control and C− is the negative, both represented in gray. The dashed line at 80% represents the relative cell viability value below which the sample extracts are deemed cytotoxic.

The cytotoxicity assay of the polymeric and composite scaffolds, with both doped and undoped BGs (Figure 5), shows that for all tested scaffold extract concentrations, relative cell viability remains above 80%. This indicates the absence of cytotoxic effects at an extract concentration of 100 mg/mL. The only exception, considering the error bars, was the 10% CuBG/PCL scaffold at the highest concentration, which exhibited slight cytotoxicity.

Given the previously reported cytotoxic effects of 1 mol% Cu-doped BG [43,44], this reduction in cell viability is more likely due to a decrease in cell proliferation during the 48-h incubation period rather than direct cell death. However, the Cu concentration can be optimized to ensure that the Cu^2+^ released from the scaffolds remains below 10 mg/L, as suggested by Wang et al. [40].

The results suggest that all tested scaffolds show potential for bone tissue engineering applications, provided that the extracellular fluid in contact with them—in either an in vivo or an in vitro model—has a high renewal rate. This would prevent local concentrations of leachates from reaching levels at which cytotoxic effects begin to appear.

#### 3.4.2. Cell Adhesion and Proliferation

**Figure 6:**
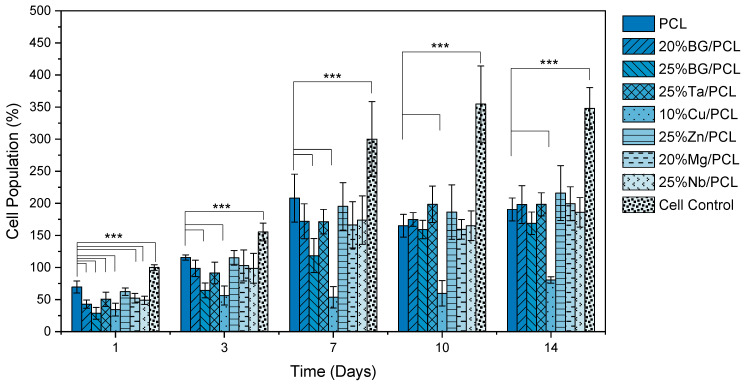
Cell relative population of Saos-2 cells cultured for 14 days on all scaffolds. Population values are normalized to the mean of the cell control values on day 1 for each biological replicate. The vertical lines denote the standard deviation of the mean. Statistical significance is indicated by ***, where *p* ˂ 0.005, comparing the PCL scaffold to other samples and the cell control.

One of the main objectives of this work is to identify the most suitable doping agent for a BG-reinforced PCL scaffold for bone regeneration. Therefore, in the second stage, all scaffolds with TIIs were tested. The results are presented in Figure 6.

The results indicate that cells adhered to all tested scaffolds. However, the incorporation of 45S5 BG at 20% and 25% significantly decreased cell adhesion compared to PCL scaffolds, and this effect is dose-dependent, as the 25% BG/PCL scaffold exhibited a significantly lower adhesion value than the 20% BG/PCL scaffold. Regarding the doped BG scaffolds, the most notable variations in behavior were observed in the 10% Cu/PCL sample, which demonstrated adverse effects on both cell adhesion and population evolution, and in the 25% Zn/PCL scaffold, which was the only one that exhibited cell adhesion levels comparable to the PCL scaffold.

All composite scaffolds, except for 10% Cu/PCL, showed an increase in cell population similar to the PCL scaffold. A comparable result to the 10% Cu/PCL scaffold was observed with Cu-MBG in a nanofibrillated cellulose (NFC) composite membrane and aerogel, where only the composite aerogel NFC:MBGSi75Cu5 with a 10:1 ratio was not cytotoxic. However, similar to the present findings, it did not enhance cell proliferation [40].

**Figure 7:**
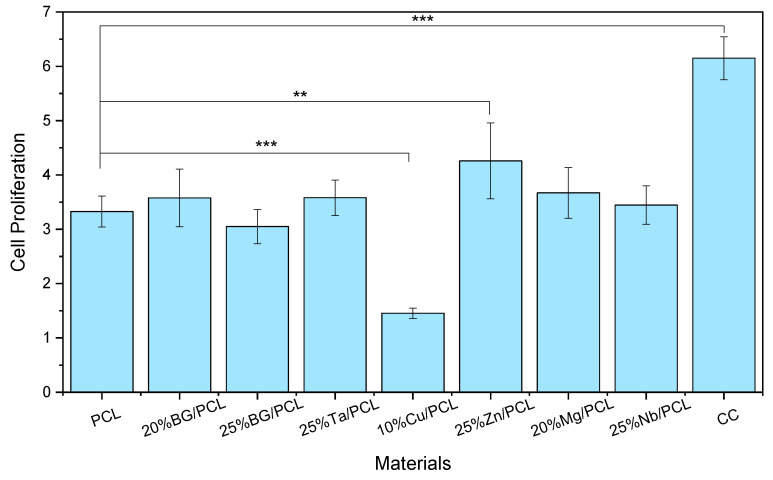
Evaluation of cell proliferation for the polymeric and doped BGs composite scaffolds is determined by the ratio of the cell population on day 7 to that on day 1. The results of the statistical significance tests between PCL and the other scaffolds are represented with ** *p* < 0.01 and *** *p* < 0.005.

The evolution of the cell population in all samples indicates cell proliferation over time; however, each sample exhibited different cell growth rates. Therefore, based on the previously presented results, the proliferation rates were determined for each sample (Figure 7).

The analysis of proliferation rates shows that the 10% Cu/PCL scaffold significantly decreased proliferation compared to all other samples in this study. In contrast, the 25% Zn/PCL scaffold was the only sample that demonstrated enhanced proliferation compared to the PCL scaffolds. Among the doped BG scaffolds, with the exception of Cu, all samples exhibited similar proliferation rates, with no significant variations. However, a slight decrease was observed when comparing Nb to Zn (*p* < 0.05).

The enhancement of cell proliferation in cultures containing Nb-doped materials has been previously reported by Obata et al. [70] and Lopes et al. [71]. In the study by Obata et al., calcium phosphate invert glasses containing Nb_2_O_5_ promoted MC3T3-E1 cell proliferation, with the highest effect observed in glasses doped with 7 mol% Nb [70]. Similarly, Lopes et al. reported that Nb-containing 45S5 BG significantly enhanced cell proliferation after four days of treatment with 1 mol% Nb [71].

The composite sample with Ta-doped BG was expected to promote higher proliferation, as previous studies on cements containing ZnO and Ta_2_O_5_ showed a significant enhancement (fourfold increase compared to the control) in fibroblast cell populations after seven days [72]. Thus, the combination of Ta- and Zn-doped BGs could potentially lead to an improved composite scaffold.

#### 3.4.3. ALP Activity

**Figure 8:**
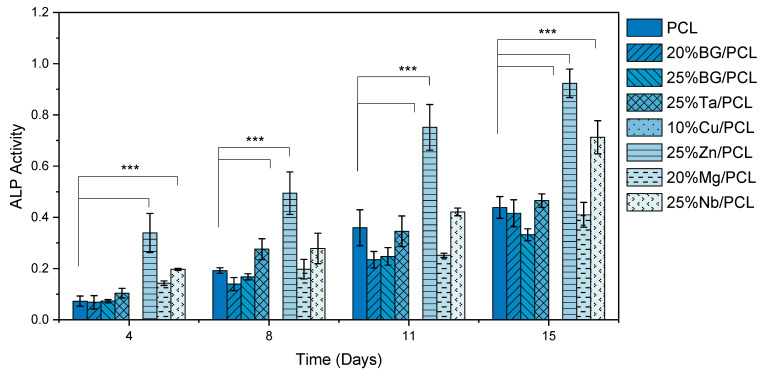
Relative ALP activity of osteoblast-like cells on all tested scaffolds for 15 days, normalized to the previous day population. The results of the statistical significance tests are represented with ***, where *p* < 0.005 for comparing the PCL scaffold to the other samples.

Analyzing the ALP production results displayed in Figure 8 revealed significant differences between the BG concentrations used and the doping ions.

The incorporation of BG into the PCL scaffolds altered the ALP activity of osteoblast-like cells. This effect is concentration-dependent, as 45S5 BG at 20% and 25% presented similar results before day 15, at which point 20%BG demonstrated a significant enhancement in ALP activity (*p* ˂ 0.05). The passivation process implemented prior to scaffold production improved the cellular response. In a previous study [49], PCL:BG scaffolds did not induce any ALP activity; however, with passivation, the same composition exhibited almost the same ALP activity as the pure PCL scaffold at the 4-day time point.

Overall, the 25% Zn/PCL scaffold exhibited the highest ALP activity at all time points, whereas the composite containing 10% Cu did not support the production of any ALP. This significant difference in ALP production between Zn- and Cu-doped BG was previously reported by Westhauser et al. [73]. The absence of ALP production in the Cu-containing sample could be attributed to an excessive concentration of Cu-doped BG. Further passivation of this BG before its incorporation into the composites might mitigate this deleterious effect. Notably, the incorporation of Cu into composites has already been shown to enhance various biomaterials. For instance, Cu-containing BG/eggshell membrane composites have been found to enhance VEGF and HIF-1α expression [41], and collagen-Cu-doped BG scaffolds (CuBG-CS) have demonstrated improved osteogenesis and angiogenesis in vitro, along with antibacterial properties and enhanced angiogenic and osteogenic responses in a chick embryo in vivo model [42].

Furthermore, the 25% Nb/PCL sample exhibited significantly enhanced ALP activity compared to the PCL scaffold. This behavior is similar to that observed in calcium phosphate invert glasses containing Nb_2_O_5_, where an upregulation of ALP expression was reported after 5 and 7 days for glasses doped with 3 and 7 mol% Nb [70].

In the final data set, after 15 days of culture, the 20% BG, 25% Ta, and 20% Mg samples exhibited ALP activity comparable to that of the PCL scaffold (0.44 ± 0.04). Furthermore, the scaffolds containing 25% Zn and 25% Nb enhanced ALP expression, yielding better results compared to all other scaffolds. Since the experiments used only osteoblast-like cells from the Saos-2 osteosarcoma cell line, future research should include other human cell types, such as mesenchymal stem cells or osteoblast precursors. To better evaluate the scaffolds’ osteoconductive and osteoinductive properties, a broader range of osteogenic markers—including RUNX2, COL1, and OCN—should also be assessed.

#### 3.4.4. Immunofluorescence Staining

Immunofluorescence imaging is a simple and effective method for studying the morphology, structure, size, and distribution of cells on sample surfaces. To investigate cell morphology after 15 days of culture on scaffolds, human osteoblast-like cells (Saos-2) were stained with phalloidin and DAPI to label the actin cytoskeleton and cell nuclei, respectively. The immunofluorescence images of the cell cultures are shown in Table 2.

Fluorescence images of the cell cultures confirmed that osteoblast-like cells adhered, proliferated, and populated all the assessed composite scaffolds over 15 days. The 10% CuBG/PCL scaffolds exhibited the lowest cell density, with only a few stained cells observed, primarily aggregated in small clusters. In all scaffolds, cell distribution appeared even, with cells infiltrating throughout the scaffolds and forming a relatively uniform distribution.

## 4. Conclusions

PCL-BG composite scaffolds, incorporating both doped and undoped BG, were successfully fabricated via solvent casting, hot pressing, and salt leaching, and their in vitro response was subsequently evaluated. The composite scaffolds exhibited a uniform morphology and a homogeneous BG distribution throughout the structure. Mechanical properties improved with increasing ceramic content, regardless of whether doped or undoped BG was used. All scaffolds were found to be safe for use as functional scaffolds. The passivation of BG powders prior to composite synthesis enhanced cell responses and enabled the incorporation of higher BG concentrations in the scaffold. However, even at lower concentrations, Cu-doped BG negatively affected cell adhesion, proliferation, and ALP production. Among the scaffolds tested, the 25% Zn/PCL scaffold, which contains 4 mol% Zn-doped BG, demonstrated the best cell proliferation and ALP activity.

Future studies should investigate the fabrication of composite scaffolds incorporating BG doped with Zn and BG doped with another TII or BG co-doped with Zn and additional TIIs. This would allow the study of possible synergistic effects of several doping TIIs in bone regeneration. For example, zinc activates osteogenic signaling pathways, promoting osteogenic differentiation and mineralization, while niobium has been shown to enhance cell adhesion, proliferation, and early matrix formation, possibly by promoting stable protein adsorption. Their combination could lead to the development of advanced biomaterials that not only support bone cell activity but also improve implant integration and accelerate tissue regeneration through complementary biological and surface-mediated mechanisms. These investigations should also include assessments using multiple cell types and in vivo evaluations.

## Figures and Tables

**Table 1:** Solutions for scaffold synthesis.

Sample Name	PCL	NaCl	BG	BG/PCL Ratio
PCL	10%	5%	-	0%
20BG			45S5 BG	20%
25BG			45S5 BG	25%
25Ta			4 mol% Ta doped BG	25%
10Cu			1 mol% Cu doped BG	10%
25Zn			4 mol% Zn doped BG	25%
20Mg			4 mol% Mg doped BG	20%
25Nb			4 mol% Nb doped BG	25%

**Table 2:** Immunofluorescence images for different samples (PCL, 20% and 25% BG/PCL, 25% Ta/PCL, 10% Cu/PCL, 25% Zn/PCL, 20% Mg/PCL and 25% Nb/PCL), where DAPI, phalloidin and the merged field are presented in the different columns.

	DAPI	Phaloidin	Merge
PCL	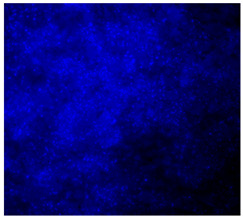	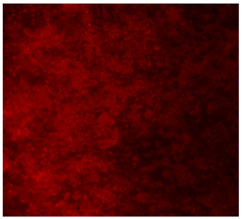	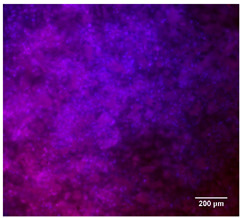
20%BG/PCL	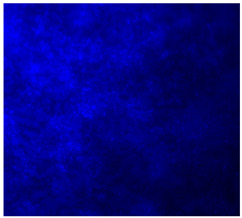	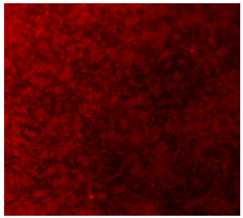	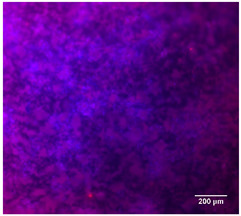
25%BG/PCL	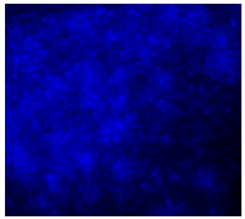	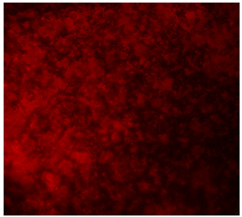	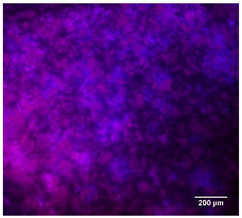
25%Ta/PCL	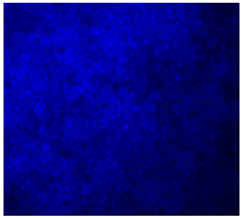	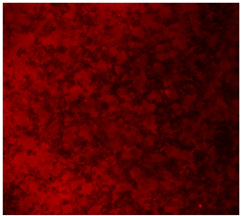	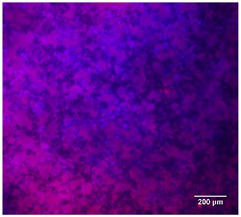
10%Cu/PCL	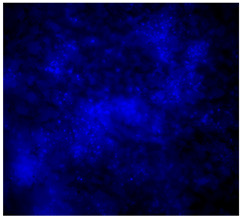	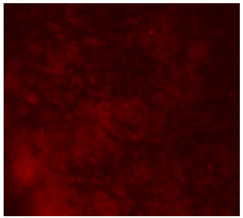	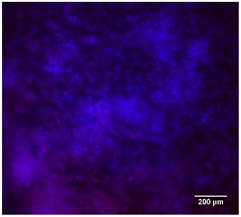
25%Zn/PCL	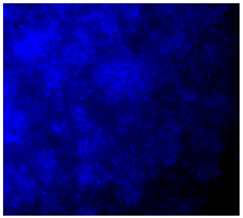	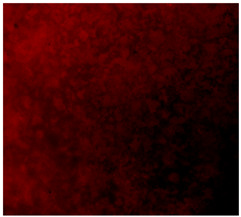	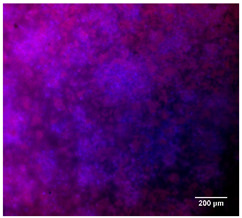
20%Mg/PCL	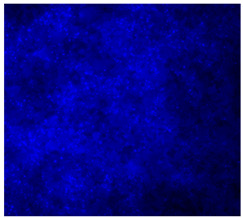	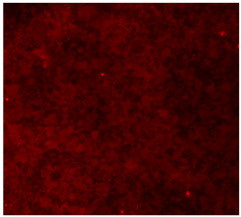	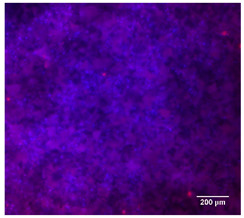
25%Nb/PCL	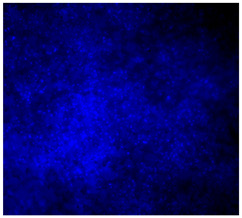	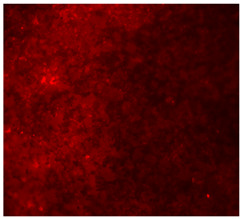	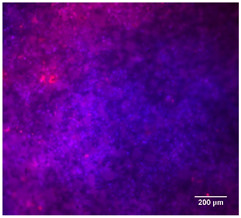
Cell Control	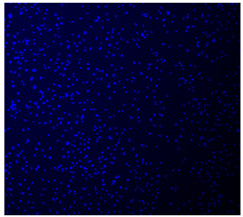	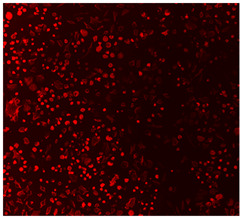	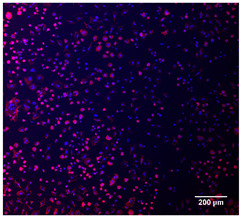

## Data Availability

The raw data supporting the conclusions of this article will be made available by the authors on request.

## Abbreviations

The following abbreviations are used in this manuscript:

ALP	Alkaline phosphatase
ATR-FTIR	Attenuated total reflectance Fourier-transform infrared
BG	Bioactive glass
Col I	Collagen type I
PRF	Platelet-rich fibrin
SEM/EDS	Scanning electron microscopy/energy dispersive X-ray spectroscopy
MBG	Mesoporous bioactive glass
NFC	Nanofibrillated cellulose
OCN	Osteocalcin
PBS	Phosphate-buffered saline
PCL	Polycaprolactone
PDLSCs	Periodontal ligament stem cells
PRF	Platelet-rich fibrin
RUNX2	Runt-related transcription factor 2
TII	Therapeutic inorganic ions
